# Methylation Profiling of Medulloblastoma in a Clinical Setting Permits Sub-classification and Reveals New Outcome Predictions

**DOI:** 10.3389/fneur.2020.00167

**Published:** 2020-03-20

**Authors:** Musa Alharbi, Nahla Mobark, Yara Bashawri, Leen Abu Safieh, Albandary Alowayn, Rasha Aljelaify, Mariam AlSaeed, Amal Almutairi, Fatimah Alqubaishi, Ebtehal AlSolme, Maqsood Ahmad, Ayman Al-Banyan, Fahad E. Alotabi, Jonathan Serrano, Matija Snuderl, May Al-Rashed, Malak Abedalthagafi

**Affiliations:** ^1^Department of Paediatric Oncology Comprehensive Cancer Centre, King Fahad Medical City, Riyadh, Saudi Arabia; ^2^Department of Biostatistics, Research Centre, King Fahad Medical City, Riyadh, Saudi Arabia; ^3^Genomics Research Department, Saudi Human Genome Project, King Fahad Medical City and King Abdulaziz City for Science and Technology, Riyadh, Saudi Arabia; ^4^Department of Neuroscience, King Fahad Medical City, Riyadh, Saudi Arabia; ^5^Department of Pathology, NYU Langone Medical Center, New York, NY, United States; ^6^Department of Clinical Laboratory Sciences, College of Applied Medical Sciences, King Saud University, Riyadh, Saudi Arabia

**Keywords:** medulloblastoma, methylation, non-glial, pediatric, neuro-oncology

## Abstract

Medulloblastoma (MB) is the most common childhood malignant brain tumor and is a leading cause of cancer-related death in children. DNA methylation profiling has rapidly advanced our understanding of MB pathogenesis at the molecular level, but assessments in Saudi Arabian (SA)-MB cases are sparse. MBs can be sub-grouped according to methylation patterns from FPPE samples into Wingless (WNT-MB), Sonic Hedgehog (SHH-MB), Group 3 (G3), and Group 4 (G4) tumors. The WNT-MB and SHH-MB subgroups are characterized by gain-of function mutations that activate oncogenic cell signaling, whilst G3/G4 tumors show recurrent chromosomal alterations. Given that each subgroup has distinct clinical outcomes, the ability to subgroup SA-FPPE samples holds significant prognostic and therapeutic value. Here, we performed the first assessment of MB-DNA methylation patterns in an SA cohort using archival biopsy material (FPPE *n* = 49). Of the 41 materials available for methylation assessments, 39 could be classified into the major DNA methylation subgroups (SHH, WNT, G3, and G4). Furthermore, methylation analysis was able to reclassify tumors that could not be sub-grouped through next-generation sequencing, highlighting its superior accuracy for MB molecular classifications. Independent assessments demonstrated known clinical relationships of the subgroups, exemplified by the high survival rates observed for WNT tumors. Surprisingly, the G4 subgroup did not conform to previously identified phenotypes, with a high prevalence in females, high metastatic rates, and a large number of tumor-associated deaths. Taking our results together, we demonstrate that DNA methylation profiling enables the robust sub-classification of four disease sub-groups in archival FFPE biopsy material from SA-MB patients. Moreover, we show that the incorporation of DNA methylation biomarkers can significantly improve current disease-risk stratification schemes, particularly concerning the identification of aggressive G4 tumors. These findings have important implications for future clinical disease management in MB cases across the Arab world.

## Introduction

Medulloblastoma (MB) is the most common malignant brain tumor in children, accounting for ~12% of childhood cancer deaths around the globe (1–5). Over the last 10 years, integrative genomics has rapidly advanced our understanding of the molecular mechanisms governing MB pathogenesis, revealing the syndrome to be more heterogeneous than previously predicted (6–18).

Advancements in cancer genomics and genome-wide transcription profiling now show that MBs comprise at least four molecular subgroups, termed Wingless (WNT-MB: mutations in CTNNB1, DDX3X, Chromatin-remodeling genes, and TP53), Sonic Hedgehog (SHH-MB: mutations in PTCH1, SMO, SUFU, TERT promoter, and Chromatin-remodeling genes), Group 3 (G3: mutations in SMARCA4, chromatin-remodeling genes, and genes of the TGF-β pathway), and Group 4 (G4: chromatin-remodeling genes) (12). The WNT-MB and SHH-MB subgroups are characterized by gain-of-function mutations that activate oncogenic cell signaling (10, 13, 18, 19). G3 and G4 have a low incidence of recurring mutations but show recurrent chromosomal alterations (18). These distinct genetic features lead to diverse clinical outcomes (5, 7, 18, 20). Patients with WNT-MB have an excellent prognosis with current therapy schemes (5-years event-free survival ≥90%) and are considered for the controlled reduction of treatment to minimize radiation and chemotherapy exposure (21–25). The prognoses of SHH-activated MBs are less favorable; SHH-MBs have an intact blood–brain barrier (BBB) and are less responsive to chemotherapy compared to WNT-MBs (22, 26, 27). Many SHH-MBs are age-dependent, with those aged ≥17 months more likely to harbor SMO and PTCH1 mutations (18, 28–32). Subgroup-driven clinical trials are currently underway to assess the efficacy of SHH pathway inhibitors such as vismodegib at diagnosis or in recurrent or refractory SHH-activated tumors.

Patients with non-WNT G3 tumors have an unfavorable prognosis, particularly if associated with MYC amplifications (33), with ≥50% of cases metastatic at the time of diagnosis (18, 27). G3 MBs are more common in males and infants and currently lack defined precision therapeutics. In contrast, G4 patients (also known as glutamatergic) are the most common molecular MB subgroup (20, 34–37) and show excellent survival with current standard-of-care treatment (2, 19, 38–40).

Neuro-epithelial brain tumors are thought to affect younger populations in Saudi Arabia (SA) compared to Western countries; MBs are the second most prevalent tumors (~13.3%) after Glioblastoma multiforme (~32%) (41). Despite advances in the use of complementary molecular genetic techniques in SA, the molecular events in SA-MB cases have not been characterized, and there is a lack of validated prognostic biomarkers. In this regard, studies assessing the DNA methylation profiles of adult brain tumors have demonstrated great promise in sub-classifying the disease and predicting clinical outcomes (42, 43). However, the methylation events in MBs have been restricted to specific genes and small cohorts, and its wider role in MB, specifically in the Arab world, remains poorly characterized.

In this study, we report the first examination of MB-DNA methylation patterns in SA using an extensive primary tumor cohort (*n* = 49). We assessed DNA methylation patterns in archival biopsy material (FPPE) to sub-classify SA-MBs and to explore the applicability of such testing to clinical applications. We herein establish methylation events as clinically useful biomarkers and demonstrate how their incorporation into current risk-stratification schemes could significantly improve the accuracy of survival predictions in SA. This holds potential for future precision therapeutic approaches aimed at improving the outcomes of afflicted SA-MB patients.

## Materials and Methods

### Patient Material

Both patient material and clinical data (*n* = 49) were obtained from the KFMC according to protocols approved by the institutional review board. Tumors were histopathologically re-assessed according to the 2016 WHO classifications. Areas of high tumor cell content (≥70%) were selected for DNA extraction. We collected essential demographic and disease-specific characteristics from the patient's electronic medical charts and radiology images to assess the extent of tumor resection. Information on neurosurgical management was obtained from operative records and standardized neurosurgical reports. Archived pathology specimens were reviewed by a board-certified neuropathologist (MA). All relevant ethical regulations were followed.

### DNA Methylation Profiling of the Saudi MB Cohort

The 450 k or EPIC (850 k) methylation array was used to obtain genome-wide DNA methylation profiles for FFPE tumor samples, according to the manufacturer's instructions (Illumina). To investigate sample stability, samples were assessed using the successor Methylation BeadChip (EPIC) array or whole-genome bisulfite sequencing. Established molecular characteristics of the WNT subgroup (CTNNB1 mutations, chromosome 6 loss), MYC and MYCN amplifications, and chromosome 17 status were assessed as previously described (13, 21–24, 27). Each MB subgroup was assessed by immunohistochemistry and mRNA expression signature assays. Methylation array processing was performed on the 450 k array to obtain genome-wide DNA methylation profiles for tumor samples. Data were generated from formalin-fixed paraffin-embedded (FFPE) tissue samples. A total of 250 ng of DNA was used for all FFPE tissues. On-chip quality metrics of all samples were controlled. Copy-number variation (CNV) analyses from the 450 k methylation datasets were performed using the conumee Bioconductor package version 1.3.0. Control samples displaying a balanced copy-number profile from both male and female donors were used for normalization.

### Bioinformatics and Statistical Analyses

Array data analysis was performed in R version 3.2.0 34, using a number of packages from Bioconductor and other repositories. A Random Forest classifier that was compatible with both 450 k and EPIC platforms was generated that calculated the class probabilities from Random Forest scores. Data (idat files) were uploaded to the Classifier (www.molecularneuropathology.org). Following the upload, the classification result was returned automatically. All datasets were submitted to the GEO database (GSE142627 https://www.ncbi.nlm.nih.gov/geo/query/acc.cgi?acc=GSE142627). For survival analysis, data were right-censored at 3 years. Overall survival (OS) and progression-free survival (PFS) were analyzed by the Kaplan-Meier (KM) method. *P*-values were found using the log-rank test. All statistical analyses were performed using SPSS 25.0 software (SPSS Inc., Chicago, IL, USA). A two-tailed *p*-value of 0.05 was considered significant.

## Results

### Patient Demographics

**Figure 1:**
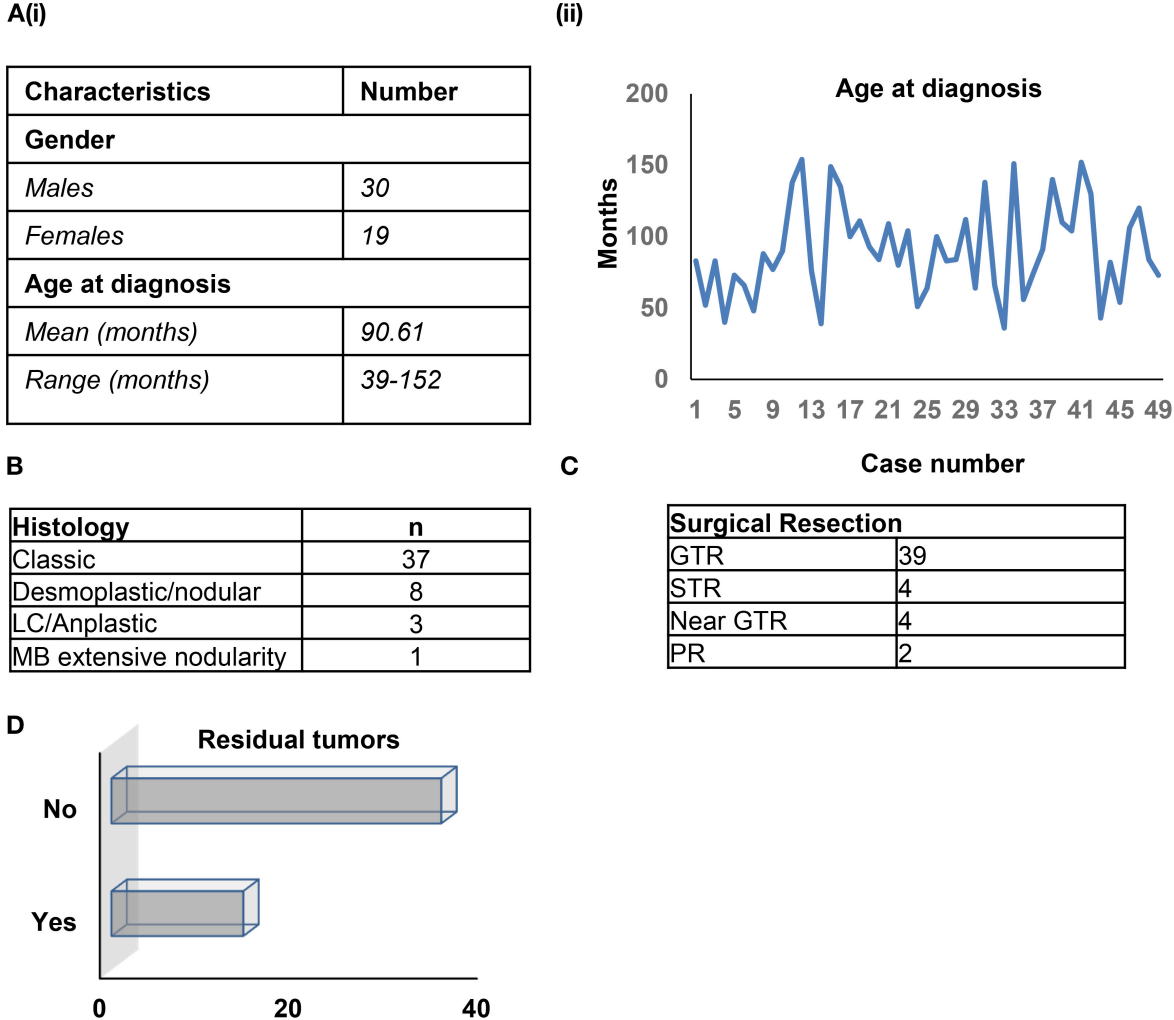
**(A–D)** Patient characteristics.

Forty-nine patients were assessed (30 males, 19 females) with a median age at diagnosis of 90.61 months (range: 39–152 months). Histopathological assessment by a board-certified neuropathologist (MA) for all cases confirmed medulloblastoma, grade IV, using 2016 WHO criteria (Figures 1A,B). The majority of diagnoses (37/49 cases) had a classic histology; 8/49 patients were classified with desmoplastic/nodular tumors, 3/49 had LC/anaplastic tumors, and a single patient showed extensive nodularity (Figure 1B). Amongst the patients, 39/49 had Gross Total Resection (GTR), of whom 7/39 had relapse/progression (Figure 1C). In total, 4/49 patients received Sub-Total Resection (STR, relapse in 2/4 patients), 4/49 received near GTR (relapse in 2/4 patients), and 2/49 received Partial Resection (PR, relapse in 1/2 patients) (Figure 1C). Nine residual tumors were observed after surgery/radiation therapy (Figure 1D). The average radiation dose was 29.341 CSI, with an average radiation boost of 27.75 CSI, which did not differ across the subgroups. All patients, excluding a single patient, received the same chemotherapeutic regimen. Medulloblastoma DNA methylation subgroups were closely related in the SA cohort. All patient characteristics have been summarized in Supplementary Table 1.

**Figure 2:**
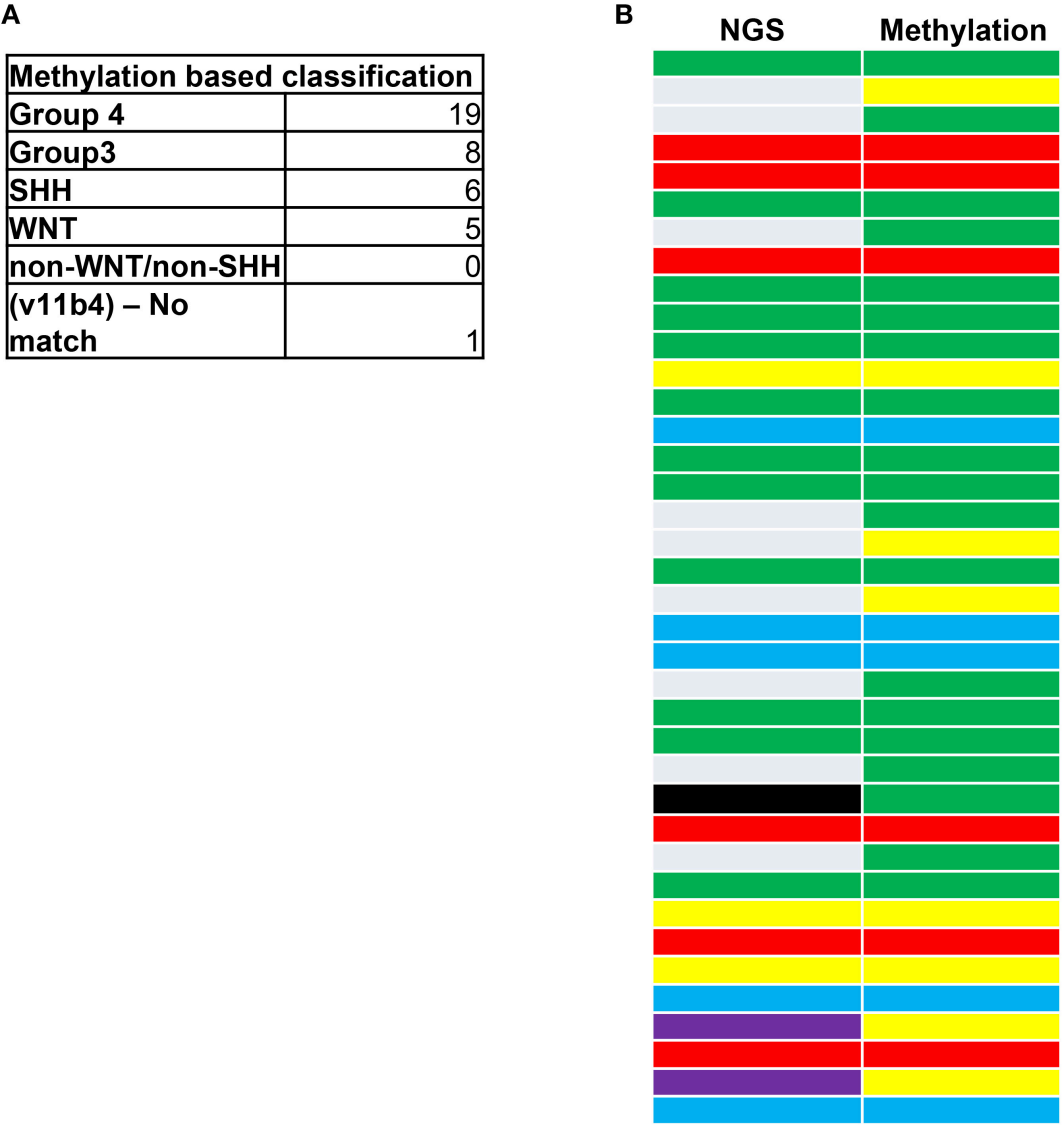
**(A)** Medulloblastoma next-generation sequencing (NGS) and DNA methylation profiling. **(B)** Classifications are shown according to the accepted coding for MB tumors: G3 (yellow), G4 (green), SHH (red), WNT (blue), and non-WNT/SHH (gray). A sample not classified by NGS (black) was classed as a G4 tumor through methylation analysis. Purple represents G3/G4 subgrouping by NGS that were subclassed as G3 tumors through methylation profiling. The correlation between NGS and methylation status was 68.42%. A total of eight samples could not be classified for methylation subgrouping due to insufficient sample material.

We next examined whether the SA cohort could be sub-classified by its DNA methylation patterns in archival diagnostic FFPE samples (11, 12, 18, 44–46). From next-generation sequencing (NGS) assessments, 45/49 tumors had been previously assigned to known WNT, SHH, G3,G4, and non-WNT-SHH subgroups (Figure 2A) using the NGS method and classification criteria as previously described (47, 48). Of the tumors, 15 were non-WNT-SHH, 1 failed due to sample quality, and 1 was unclassifiable. To assess the efficacy of methylation sub-grouping, DNA methylation events were then profiled using the Illumina Infinium HumanMethylation450 BeadChip (450 k) arrays using established molecular characteristics of DNA methylation events (42, 43). This permitted repeat analysis of the cohort to compare the NGS and methylation methods and to reclassify the “non-WNT-SHH cases” observed from NGS analysis.

Materials were available for 41/49 cases, all of which were classified, excluding a single sample that we deemed as a “non-match.” Thus, 40/41 tumors could be sub-grouped (Figure 2A). In total, 68.42% of the tumors were in agreement between NGS and methylation status (Figure 2B). The fact that DNA methylation can control several genes that could contribute to MB progression may have accounted for the tumors that did not correlate. Of these, nine of the non-WNT-SHH cases from NGS assessments were classified by methylation as either G3 (five cases) or G4 (four cases). All WNT and SHH groups were matched by the two methods. Of note, a case that failed in NGS passed the methylation assay as a G4 tumor. This highlighted the accuracy for MB subgrouping to classify tumors that were deemed unambiguous molecular subgroups that could be neither assigned nor classified by the molecular neuropathology classifier. The high prevalence of G4 tumors was consistent with reports of this subtype having the highest occurrence in other cohorts (20, 34–37).

### Methylation Subgroups Show Distinct Clinical and Pathological Features

**Figure 3:**
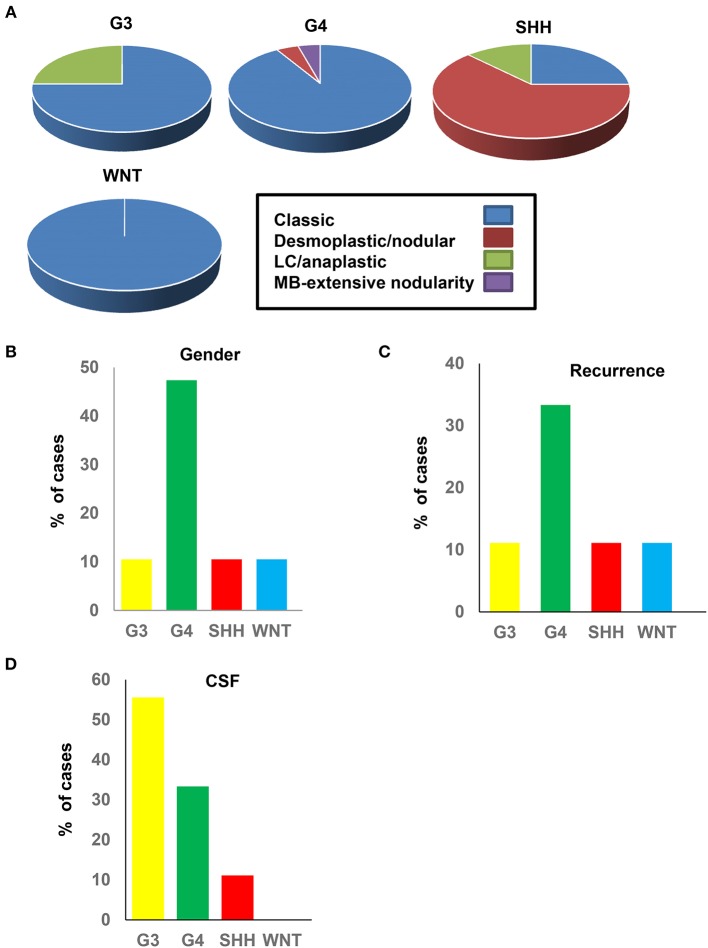
Clinical and pathological data of the medulloblastoma methylation subgroups. **(A)** Histological classification in each subtype. **(B)** Gender percentage in each subtype. **(C)** Percentage of recurrence in each subtype. **(D)** CSF percentage dissemination in each subtype.

We next investigated whether the clinico-pathological features of the methylation subgroups were consistent with those reported in previous cohorts (12, 18, 46). SHH-MB subgroups showed significant enrichment for desmoplastic/nodular (DN) pathology, which was consistent with previous studies (24) (Figure 3A). G3, G4, and WNT tumors were predominantly of classic histology (Figure 3A). All subgroups were predominantly males, excluding G4 tumors, which approached an uncharacteristic 50:50 male/female ratio (Figure 3B). G3 MBs are more common in males, consistent with what was observed in our cohort, but the high prevalence of female G4 sub-grouped patients has not, to our knowledge, been reported previously and may be unique to the SA cohort. WNT and SHH subgroups showed favorable outcomes, as previously reported from IHC studies, showing low levels of tumor recurrence (Figure 3C). Recurrence was most prevalent in G4 groups, which again was surprising. Dissemination via the *cerebrospinal fluid* (*CSF*) is indicative of tumor malignancy and was most prevalent in G3, followed by G4 (Figure 3D). Whilst the aggressive nature of G3 tumors would be expected (particularly when associated with MYC amplifications), the high metastatic rates in G4 tumors were unexpected and again may represent a unique feature of the SA cohort.

### Methylation Subgroups Can Dictate Survival Outcomes for SA-MB Patients

**Figure 4:**
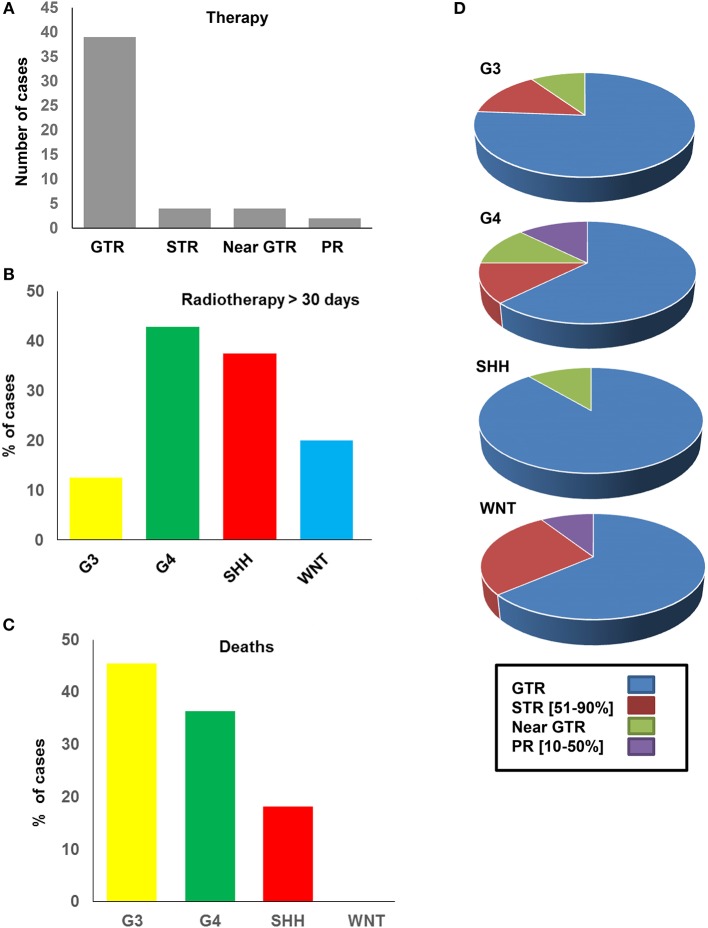
Therapeutic strategy and outcomes of the SA medulloblastoma methylation subgroups. **(A)** Number of cases received GTR, STR, near GTR, and PR surgical resection. **(B)** Percentage of cases in each subtypes which received radiotherapy. **(C)** Percentage of deaths in each subtype. **(D)** Percentage of GTR, STR, near GTR, PR in each molecular subtype.

**Figure 5:**
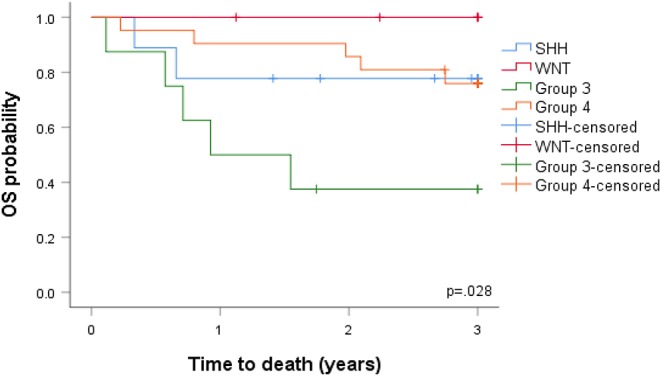
Overall survival (OS) and progression-free survival (PFS) analyzed by the Kaplan-Meier method.

In view of the changes in clinical and pathological features observed, we investigated the prognostic potential of the individual DNA methylation subgroups in our SA cohort (Figure 4). We first examined the extent of tumor resection based on post-operative imaging, which we classified as gross total (GTR), near GTR (≥90%), subtotal (STR 51–91%), or partial (PR 10–50%). Whilst most patients underwent GTR, STRs were prevalent in the WNT subgroups (Figures 4A,B). There was no obvious benefit of GTR over STR, as previously reported, but such comparisons were limited by the small sample size (49). In terms of treatment, G4 and SHH subgroups received the most prolonged radiotherapy regimens (≥30 days, Figure 4B), though the radiation doses were comparable between the groups. The percentage of deaths were, as expected, highest in the G3-MB subgroup (Figure 4C). Consistent with the CSF findings, G4 tumors showed a surprisingly high percentage of deaths, exceeding those of the SHH subgroup, which typically respond poorly to MB therapy (Figure 4D). No deaths were observed in the WNT-MB subgroup, consistent with the limited aggressiveness of these tumors. These findings were confirmed by Kaplan Meier (KM) analysis (Figure 5). The 3-years OS for the entire cohort was 0.707 (95% CI: 0.540–0.823) and the 3-years PFS was 0.685 (95% CI: 0.519, 0.804). We observed a significant difference in survival amongst the molecular subgroups from pooled analysis (*p* = 0.028). The 3-years OS for the SHH subgroup was 0.778 (95% CI: 0.365–0.939) compared to 0.375 for G3 (95% CI: 0.087–0.674) and 0.759 for G4 (95% CI: 0.670–0.975), which was again surprising. Additionally, the 3-years PFS for the SHH subgroup was 0.778 (95% CI: 0.470–0.939) compared to 0.375 for G3 (95% CI: 0.087–0.674) and 0.714 for G4 (95% CI: 0.472–0.860) (*p* = 0.035 across all four subgroups). No events were observed in the WNT group. It is therefore advised that patients with WNT-MB in SA be considered for the controlled reduction of treatment to minimize radiation and chemotherapy side effects (21–25).

## Discussion

Medulloblastoma (MB) is the most common pediatric brain tumor in SA, with a high incidence amongst children aged ≤ 5 years (41). Disease dissemination is an early event in MB, with up to ~40% of patients showing metastases at diagnosis, with poor survival. Metastatic disease and tumor recurrence are responsible for the low survival rates, and those who survive frequently show treatment-related adverse effects (5, 20, 35, 36, 38, 40, 50–53).

In this study, we report the first comprehensive investigation of gene-specific DNA methylation profiles in SA. Our hospital is a major referral center for pediatric oncology, and the cases represent most regions of SA. The SA MB cohort highlights the potential of epigenetics to improve disease management in the Arab world. Using cross-validated class-discovery approaches, we demonstrate that MBs in SA can be subdivided into the four major DNA methylation subgroups SHH, WNT, G3, and G4 (12, 18, 46). Independent assessments of each subgroup demonstrated a series of previously described relationships, exemplified by the excellent responsiveness and high survival of WNT patients under standard treatment protocols (13, 18, 21–25, 27, 45, 54, 55). Unique features were also identified, namely the aggressiveness of G4 tumors, leading to ~1/3 deaths. This expands our knowledge of the molecular aberrations involved in MB tumorigenesis in SA.

DNA methylation assessments allowed the robust discrimination of subgroup status in 39/41 tumors in FFPE biopsies, which were comparable to the rates of transcriptomic methods (43/49). This offered accurate distinctions in the sub-classification of SA patients from archival cohorts (33). A subset of 8/49 samples could not be classified due to insufficient material for our analysis. Thus, our methylation assessments were more accurate than the ~6% reported in previous cohorts (22, 24, 43, 55, 56). Importantly, methylation analysis permitted the classification of tumors that could not be sub-grouped through NGS assessments, highlighting its higher accuracy for MB sub-grouping.

G4 MBs are the most prevalent biological subtype, comprising ~40% of all MB tumors, which predominantly occur in those aged 3 to 16 years (22, 23, 50, 57, 58). G4 is reportedly 3-fold more frequent in males than females across all age groups, with an intermediate prognosis and 5-years survival reaching ~80% with standard therapy, although non-metastatic tumors with chromosome 11 loss have an excellent prognosis, with > 90% survival (18, 44, 59). Up to 30–40% of G4 MB patients have metastases at diagnosis and are treated as high risk (7, 49). Adults with G4 MBs have a significantly poorer prognosis compared to the SHH- or WNT-subtypes (22, 23, 57). In our cohort, the male/female ratio was close to 50:50 in the G4 subgroup, arguing against a high gender preference in the SA population. This was discordant with the male/female ratios reported in previous cohorts, in which 80–90% of G4 cases are males. The majority of G4 tumors were of classic histology, with the rest of large cell/anaplastic histology 2, consistent with previous findings (7, 10, 18, 44, 49, 59). A high number of tumors showed CSF dissemination, suggestive of metastasis. The number of deaths in the G4 subgroup was surprisingly high (36.36%), indicative of a lower prognosis that the > 90% survival reported in other cohorts (7). These results imply a more aggressive nature of G4 MBs in SA carriers that may result from both genetic and environmental factors. The discrepancies may also be, in part, due to study limitations, including our small sample size (*n* = 21 G4 tumors), lack of adjustment for disease characteristics, cancer treatment modalities, or the length of follow-up. In addition, we did not re-evaluate the cases for additional molecular diagnostics such as copy number profiling, and such analyses may further explain the unique features observed in G4-SA tumors. Future studies needed to further evaluate this observation.

Our study was performed in an all-SA cohort where all study participants underwent the same methylation-based assessments. The follow-ups were extensive, and tumor characteristics and outcome data were collected in a prospective manner. The majority of MB patients received treatment in a single cancer center with consistent treatment guidelines. This homogeneity of data collection strengthens the validity of our prognostic analysis, revealing G4 tumors in SA to represent an aggressive MB subtype.

Our cohort displayed similar features to previous studies regarding the aggressiveness of G3 tumors (23, 30, 50, 57, 58, 60). G3 and G4 MBs were more closely related than WNT and SHH and appear as non-WNT/non-SHH in the revised 2016 WHO classification, but historically these tumors are molecularly and clinically heterogeneous (10, 18, 44, 49, 50, 59, 61). G3 MBs cause ~25% of all cases of MB (16.32% in our cohort) predominantly amongst infants, with a peak diagnosis between ages 3–5 years. In our cohort, the mean age at diagnosis was 90.61 months, suggestive of delayed disease progression. The male-to-female ratio of these tumors has been reported as 1:2, with a ~60 5-years overall survival in children and a 45% 5-years overall survival in infants (7). The poor prognosis of G3 MBs is related to the young age of metastases (~50% of patients vs. 55.55% in our cohort) at diagnosis, large cell/anaplastic (LCA) histology (25% of tumors in our cohort), and MYC amplification (33). G3 MYC-amplified tumors confer an especially short survival, with only 1 in 5 patients surviving 5 years. G3 MBs also rarely recur at the original tumor site, consistent with the ≤ 10% recurrence observed in this study. G3 tumor metastases are frequent, but the rates do not dictate survival. Significant differences in survival amongst the molecular subgroups from pooled analysis (*p* = 0.028) was also evident; the 3-years OS for the SHH subgroup was 0.778 (95% CI: 0.365–0.939) compared to 0.375 for G3 (95% CI: 0.087–0.674) and 0.759 for G4 (95% CI: 0.670–0.975), highlighting the aggressive nature of G4 tumors in the cohort. The 3-years PFS for the SHH subgroup was 0.778 (95% CI: 0.470–0.939) compared to 0.375 for G3 (95% CI: 0.087–0.674) and 0.714 for G4 (95% CI: 0.472–0.860) (*p* = 0.035 across all four subgroups). Given the close association of SA G3 cohorts to those previously reported, we recommend that SA G3-MB children in the presence/absence of disease spread should be assigned to standard-risk groups to avoid under-treatment. Precision therapies are yet to be developed for G3 tumors due to our limited understanding of tumorigenesis. A consistent finding was the low number of deaths observed in WNT subgroups, which we confirmed through KM analysis (18, 25, 62). Given these low rates, we recommend that this tumor subgroup should receive reduction therapy in SA, thus avoiding unnecessary treatments that have physical consequences for patients and their families and place a substantial financial burden on SA healthcare centers. The development of rational treatment approaches should also be focused on high-risk and metastatic non-WNT/non-SHH patients to suppress the poor survival rates of these subgroups in SA. As precision therapeutics for G3 and G4 tumors improve, the addition of DNA methylation biomarkers is likely to significantly improve survival predictions at diagnosis in SA, as described for previous cohorts (63–65). Our SA cohort further highlights the essential role of the methylation array as a prognostic tool to improve clinicians' ability to manage MB patients.

## Data Availability Statement

The datasets generated for this study can be found in the All datasets submitted to GEO database (https://www.ncbi.nlm.nih.gov/geo/query/acc.cgi?acc=GSE142627).

## Ethics Statement

The studies involving human participants were reviewed and approved by King Fahad Medical City IRB 16-310. Written informed consent from the participants' legal guardian/next of kin was not required to participate in this study in accordance with the national legislation and the institutional requirements.

## Author Contributions

MAlh, NM, and MAb collected, analyzed the clinical data, and wrote part of the manuscript. MAlS, JS, and MA-R performed experiments. LA, EA, AAlo, RA, MAh, AAlm, and FA helped in the experimental studies and data analysis. MAb and AA-B collected the clinical samples. MAb designed the study, analyzed the data, and wrote the manuscript. All authors approved the final manuscript.

### Conflict of Interest

The authors declare that the research was conducted in the absence of any commercial or financial relationships that could be construed as a potential conflict of interest.

## Supplementary Material

Supplementary Table 1 Detailed clinical and molecular data of the SA MB cohort.

## References

[1] Rolland A Aquilina K. Surgery for recurrent medulloblastoma: a review. Neurochirurgie. (2019) 10.1016/j.neuchi.2019.06.008. [Epub ahead of print]. 31351079

[2] Mollashahi B Aghamaleki FS Movafagh A. The roles of miRNAs in medulloblastoma: a systematic review. J Cancer Prev. (2019) 24:79–90. 10.15430/JCP.2019.24.2.79 31360688 PMC6619858

[3] Wu X Zhou Y Li L Liang P Zhai X. Post-treatment maturation of medulloblastoma in children: two cases and a literature review. J Int Med Res. (2018) 46:4781–90. 10.1177/0300060518788251 30270802 PMC6259389

[4] Wang Y Song S Su X Wu J Dai Z Cui D. Radiation-induced glioblastoma with rhabdoid characteristics following treatment for medulloblastoma: a case report and review of the literature. Mol Clin Oncol. (2018) 9:415–8. 10.3892/mco.2018.1703 30233795 PMC6142298

[5] Thompson EM Bramall A Herndon JE Taylor MD Ramaswamy V. The clinical importance of medulloblastoma extent of resection: a systematic review. J Neurooncol. (2018) 139:523–39. 10.1007/s11060-018-2906-5 29796724

[6] C Di Pietro La Sala G Matteoni R Marazziti D Tocchini-Valentini PG Genetic ablation of Gpr37l1 delays tumor occurrence in Ptch1(+/-) mouse models of medulloblastoma. Exp Neurol. (2019) 312:33–42. 10.1016/j.expneurol.2018.11.004 30452905

[7] Waszak SM Northcott PA Buchhalter I Robinson GW Sutter C Groebner S. Spectrum and prevalence of genetic predisposition in medulloblastoma. a retrospective genetic study and prospective validation in a clinical trial cohort. Lancet Oncol. (2018) 19:785–98. 10.1016/S1470-2045(18)30242-0 29753700 PMC5984248

[8] Chen YD Zhang N Qiu XG Yuan J Yang M. LncRNA CDKN2BAS rs2157719 genetic variant contributes to medulloblastoma predisposition. J Gene Med. (2018) 20:e3000. 10.1002/jgm.3000 29314442

[9] Baocheng W Zhao Y Meng W Han Y Wang J Liu F. Polymorphisms of insulin receptor substrate 2 are putative biomarkers for pediatric medulloblastoma: considering the genetic susceptibility and pathological diagnoses. Nagoya J Med Sci. (2017) 79:47–54. 10.18999/nagjms.79.1.47 28303061 PMC5346620

[10] Shi X Wang Q Gu J Xuan Z Wu IJ. SMARCA4/Brg1 coordinates genetic and epigenetic networks underlying Shh-type medulloblastoma development. Oncogene. (2016) 35:5746–58. 10.1038/onc.2016.108 27065321

[11] Pietsch T Haberler C. Update on the integrated histopathological and genetic classification of medulloblastoma - a practical diagnostic guideline. Clin Neuropathol. (2016) 35:344–52. 10.5414/NP300999 27781424 PMC5094373

[12] Skowron P Ramaswamy V Taylor DM. Genetic and molecular alterations across medulloblastoma subgroups. J Mol Med. (2015) 93:1075–84. 10.1007/s00109-015-1333-8 26350064 PMC4599700

[13] Goschzik T Zur Muhlen A Kristiansen G Haberler C Stefanits H Friedrich C. Molecular stratification of medulloblastoma: comparison of histological and genetic methods to detect Wnt activated tumours. Neuropathol Appl Neurobiol. (2015) 41:135–44. 10.1111/nan.12161 24894640

[14] Brown AL Lupo PJ Okcu MF Lau CC Rednam S Scheurer EM. SOD2 genetic variant associated with treatment-related ototoxicity in cisplatin-treated pediatric medulloblastoma. Cancer Med. (2015) 4:1679–86. 10.1002/cam4.516 26400460 PMC4673994

[15] Jenkins NC Kalra RR Dubuc A Sivakumar W Pedone CA Wu X. Genetic drivers of metastatic dissemination in sonic hedgehog medulloblastoma. Acta Neuropathol Commun. (2014) 2:85. 10.1186/s40478-014-0085-y 25059231 PMC4149244

[16] Wu X Northcott PA Dubuc A Dupuy AJ Shih DJ Witt H. Clonal selection drives genetic divergence of metastatic medulloblastoma. Nature. (2012) 482:529–33. 10.1038/nature10825 22343890 PMC3288636

[17] Onvani S Terakawa Y Smith C Northcott P Taylor M Rutka J. Molecular genetic analysis of the hepatocyte growth factor/MET signaling pathway in pediatric medulloblastoma. Genes Chromosomes Cancer. (2012) 51:675–88. 10.1002/gcc.21954 22447520

[18] Kool M Korshunov A Remke M Jones DT Schlanstein M Northcott PA. Molecular subgroups of medulloblastoma: an international meta-analysis of transcriptome, genetic aberrations, and clinical data of WNT, SHH, Group 3, and Group 4 medulloblastomas. Acta Neuropathol. (2012) 123:473–84. 10.1007/s00401-012-0958-8 22358457 PMC3306778

[19] Mangum R Varga E Boue DR Capper D Benesch M Leonard J. SHH desmoplastic/nodular medulloblastoma and Gorlin syndrome in the setting of Down syndrome: case report, molecular profiling, and review of the literature. Childs Nerv Syst. (2016) 32:2439–46. 10.1007/s00381-016-3185-0 27444290

[20] Bautista F Fioravantti V de Rojas T Carceller F Madero L Lassaletta A. Medulloblastoma in children and adolescents: a systematic review of contemporary phase I and II clinical trials and biology update. Cancer Med. (2017) 6:2606–24. 10.1002/cam4.1171 28980418 PMC5673921

[21] Bendelsmith CR Skrypek MM Patel SR Pond DA Linabery AM Bendel EA. Multiple pilomatrixomas in a survivor of WNT-activated medulloblastoma leading to the discovery of a germline APC mutation and the diagnosis of familial adenomatous polyposis. Pediatr Blood Cancer. (2018) 65:e26756. 10.1002/pbc.26756 28792655

[22] Cambruzzi E. Medulloblastoma, WNT-activated/SHH-activated: clinical impact of molecular analysis and histogenetic evaluation. Childs Nerv Syst. (2018) 34:809–15. 10.1007/s00381-018-3765-2 29582169

[23] Geron L Salomao KB Borges KS Andrade AF Correa CAP Scrideli CA. Molecular characterization of Wnt pathway and function of beta-catenin overexpression in medulloblastoma cell lines. Cytotechnology. (2018) 70:1713–22. 10.1007/s10616-018-0260-2 30374857 PMC6269366

[24] Goschzik T Schwalbe EC Hicks D Smith A Muehlen AZ Figarella-Branger D. Prognostic effect of whole chromosomal aberration signatures in standard-risk, non-WNT/non-SHH medulloblastoma: a retrospective, molecular analysis of the HIT-SIOP PNET 4 trial. Lancet Oncol. (2018) 19:1602–16. 10.1016/S1470-2045(18)30532-1 30392813 PMC6262170

[25] Lastowska M Trubicka J Karkucinska-Wieckowska A Kaleta M Tarasinska M Perek-Polnik M. Immunohistochemical detection of ALK protein identifies APC mutated medulloblastoma and differentiates the WNT-activated medulloblastoma from other types of posterior fossa childhood tumors. Brain Tumor Pathol. (2019) 36:1–6. 10.1007/s10014-018-0331-2 30523493 PMC6514113

[26] Bhatia B Potts CR Guldal C Choi S Korshunov A Pfister S. Hedgehog-mediated regulation of PPARgamma controls metabolic patterns in neural precursors and shh-driven medulloblastoma. Acta Neuropathol. (2012) 123:587–600. 10.1007/s00401-012-0968-6 22407012 PMC3306783

[27] Bourdeaut F Miquel C Richer W Grill J Zerah M Grison C. Rubinstein-Taybi syndrome predisposing to non-WNT, non-SHH, group 3 medulloblastoma. Pediatr Blood Cancer. (2014) 61:383–6. 10.1002/pbc.24765 24115570

[28] Coni S Mancuso AB di Magno L Sdruscia G Manni S Serrao SM. Corrigendum: selective targeting of HDAC1/2 elicits anticancer effects through Gli1 acetylation in preclinical models of SHH Medulloblastoma. Sci Rep. (2017) 7:46645. 10.1038/srep46645 28429760 PMC5399352

[29] Fudyma IA Wadhwani RN. Medulloblastoma with extensive nodularity (SHH medulloblastoma). Pol J Pathol. (2017) 68:364–6. 10.5114/pjp.2017.73934 29517209

[30] Gentile G Ceccarelli M Micheli L Tirone F Cavallaro S. Functional genomics identifies Tis21-dependent mechanisms and putative cancer drug targets underlying medulloblastoma shh-type development. Front Pharmacol. (2016) 7:449. 10.3389/fphar.2016.00449 27965576 PMC5127835

[31] Liang L Coudiere-Morrison L Tatari N Stromecki M Fresnoza A Porter CJ. CD271(+) cells are diagnostic and prognostic and exhibit elevated MAPK activity in SHH medulloblastoma. Cancer Res. (2018) 78:4745–59. 10.1158/0008-5472.CAN-18-0027 29930101

[32] Merk DJ Ohli J Merk ND Thatikonda V Morrissy S Schoof M. Opposing effects of CREBBP mutations govern the phenotype of rubinstein-taybi syndrome and adult SHH medulloblastoma. Dev Cell. (2018) 44:709–24 e6. 10.1016/j.devcel.2018.02.012 29551561

[33] Aldosari N Bigner SH Burger PC Becker L Kepner JL Friedman HS McLendon ER. MYCC and MYCN oncogene amplification in medulloblastoma. A fluorescence *in situ* hybridization study on paraffin sections from the Children's Oncology Group. Arch Pathol Lab Med. (2002) 126:540–4. 11958658 10.5858/2002-126-0540-MAMOAI

[34] Doger de Speville E Kieffer V Dufour C Grill J Noulhiane M Hertz-Pannier L Neuropsychological consequences of childhood medulloblastoma and possible interventions: a review. Neurochirurgie. (2018). 10.1016/j.neuchi.2018.03.002. [Epub ahead of print]. 29716738

[35] Drissi J Affane M Elomrani A Khouchani M. Medulloblastoma in adults: report of 13 cases and literature review. Pan Afr Med J. (2015) 22:126. 10.11604/pamj.2015.22.126.7242 26889307 PMC4742019

[36] Faried A Pribadi MA Sumargo S Arifin MZ Hernowo SB. Adult medulloblastoma: a rare case report and literature review. Surg Neurol Int. (2016) 7(Suppl. 17):S481–4. 10.4103/2152-7806.185782 27512610 PMC4960923

[37] Goyal A Cajigas I Ibrahim GM Brathwaite CD Khatib Z Niazi T. Surgical treatment of intramedullary spinal metastasis in medulloblastoma: case report and review of the literature. World Neurosurg. (2018) 118:42–6. 10.1016/j.wneu.2018.06.250 29990605

[38] Liang B Feng E Wang Q Cao Y Li P Li Y. Medulloblastoma in an elderly patient: a case report and literature review. Mol Clin Oncol. (2016) 5:312–4. 10.3892/mco.2016.942 27588198 PMC4998084

[39] Murase M Saito K Abiko T Yoshida K Tomita H. Medulloblastoma in older adults: a case report and literature review. World Neurosurg. (2018) 117:25–31. 10.1016/j.wneu.2018.05.216 29883827

[40] Noiphithak R Yindeedej V Thamwongskul C. Cerebellopontine angle medulloblastoma with extensive nodularity in a child: case report and review of the literature. Childs Nerv Syst. (2017) 33:839–42. 10.1007/s00381-016-3325-6 28013334

[41] Taha MS Almsned FM Hassen MA Atean IM Alwbari AM Alharbi QK. Demographic and histopathological patterns of neuro-epithelial brain tumors in Eastern province of Saudi Arabia. Neurosciences. (2018) 23:18–22. 10.17712/nsj.2018.1.20160543 29455216 PMC6751907

[42] Miele E Valente S Alfano V Silvano M Mellini P Borovika D. The histone methyltransferase EZH2 as a druggable target in SHH medulloblastoma cancer stem cells. Oncotarget. (2017) 8:68557–70. 10.18632/oncotarget.19782 28978137 PMC5620277

[43] Northcott PA Nakahara Y Wu X Feuk L Ellison DW Croul S. Multiple recurrent genetic events converge on control of histone lysine methylation in medulloblastoma. Nat Genet. (2009) 41:465–72. 10.1038/ng.336 19270706 PMC4454371

[44] Remke M Ramaswamy V Peacock J Shih DJ Koelsche C Northcott PA. TERT promoter mutations are highly recurrent in SHH subgroup medulloblastoma. Acta Neuropathol. (2013) 126:917–29. 10.1007/s00401-013-1198-2 24174164 PMC3830749

[45] Rodriguez-Blanco J Pednekar L Penas C Li B Martin V Long J. Inhibition of WNT signaling attenuates self-renewal of SHH-subgroup medulloblastoma. Oncogene. (2017) 36:6306–14. 10.1038/onc.2017.232 28714964 PMC5680121

[46] Thompson MC Fuller C Hogg TL Dalton J Finkelstein D Lau CC. Genomics identifies medulloblastoma subgroups that are enriched for specific genetic alterations. J Clin Oncol. (2006) 24:1924–31. 10.1200/JCO.2005.04.4974 16567768

[47] AlSahlawi A Aljelaify R Magrashi A AlSaeed M Almutairi A Alqubaishi F. New insights into the genomic landscape of meningiomas identified FGFR3 in a subset of patients with favorable prognoses. Oncotarget. (2019) 10:5549–59. 10.18632/oncotarget.27178 31565188 PMC6756861

[48] Ramkissoon SH Bandopadhayay P Hwang J Ramkissoon LA Greenwald NF Schumacher SE. Clinical targeted exome-based sequencing in combination with genome-wide copy number profiling: precision medicine analysis of 203 pediatric brain tumors. Neuro Oncol. (2017) 19:986–96. 10.1093/neuonc/nox083.080 28104717 PMC5570190

[49] Thompson EM Hielscher T Bouffet E Remke M Luu B Gururangan S. Prognostic value of medulloblastoma extent of resection after accounting for molecular subgroup: a retrospective integrated clinical and molecular analysis. Lancet Oncol. (2016) 17:484–95. 10.1016/S1470-2045(15)00581-1 26976201 PMC4907853

[50] Kline CN Packer RJ Hwang EI Raleigh DR Braunstein S Raffel C. Case-based review: pediatric medulloblastoma. Neurooncol Pract. (2017) 4:138–50. 10.1093/nop/npx011 29692919 PMC5909805

[51] Raghu ALB Kandasamy J Brougham M Gallo P Sokol D Hughes AM. Delayed diffuse cerebellar swelling after resection of medulloblastoma: case report and review of literature. Childs Nerv Syst. (2017) 33:2047–9. 10.1007/s00381-017-3496-9 28664279 PMC5644696

[52] Pant I Chaturvedi S Gautam VK Pandey P Kumari R. Extra-axial medulloblastoma in the cerebellopontine angle: report of a rare entity with review of literature. J Pediatr Neurosci. (2016) 11:331–4. 10.4103/1817-1745.199477 28217158 PMC5314849

[53] Valvi S Ziegler SD. Ganglioglioma arising from desmoplastic medulloblastoma: a case report and review of literature. Pediatrics. (2017) 139:e20161403. 10.1542/peds.2016-1403 28232638

[54] Remke M Hielscher T Korshunov A Northcott PA Bender S Kool M. FSTL5 is a marker of poor prognosis in non-WNT/non-SHH medulloblastoma. J Clin Oncol. (2011) 29:3852–61. 10.1200/JCO.2011.36.2798 21911727

[55] Li YX Shao LW Jiang T Liu Y Chang Q. miR-449a is a potential epigenetic biomarker for WNT subtype of medulloblastoma. Zhonghua Bing Li Xue Za Zhi. (2017) 46:684–9. 10.3760/cma.j.issn.0529-5807.2017.10.005 29050069

[56] Nakahara Y Northcott PA Li M Kongkham PN Smith C Yan H. Genetic and epigenetic inactivation of Kruppel-like factor 4 in medulloblastoma. Neoplasia. (2010) 12:20–7. 10.1593/neo.91122 20072650 PMC2805880

[57] Ecker J Oehme I Mazitschek R Korshunov A Kool M Hielscher T. Targeting class I histone deacetylase 2 in MYC amplified group 3 medulloblastoma. Acta Neuropathol Commun. (2015) 3:22. 10.1186/s40478-015-0201-7 25853389 PMC4382927

[58] Gottardo NG Hansford JR McGlade JP Alvaro F Ashley DM Bailey S. Medulloblastoma down under 2013: a report from the third annual meeting of the international medulloblastoma working group. Acta Neuropathol. (2014) 127:189–201. 10.1007/s00401-013-1213-7 24264598 PMC3895219

[59] Merve A Dubuc AM Zhang X Remke M Baxter PA Li XN. Polycomb group gene BMI1 controls invasion of medulloblastoma cells and inhibits BMP-regulated cell adhesion. Acta Neuropathol Commun. (2014) 2:10. 10.1186/2051-5960-2-10 24460684 PMC3928978

[60] Faria CC Agnihotri S Mack SC Golbourn BJ Diaz RJ Olsen S. Identification of alsterpaullone as a novel small molecule inhibitor to target group 3 medulloblastoma. Oncotarget. (2015) 6:21718–29. 10.18632/oncotarget.4304 26061748 PMC4673298

[61] Morfouace M Cheepala S Jackson S Fukuda Y Patel YT Fatima S. ABCG2 transporter expression impacts group 3 medulloblastoma response to chemotherapy. Cancer Res. (2015) 75:3879–89. 10.1158/0008-5472.CAN-15-0030 26199091 PMC4573843

[62] Kool M Koster J Bunt J Hasselt NE Lakeman A van Sluis P Troost D. Integrated genomics identifies five medulloblastoma subtypes with distinct genetic profiles, pathway signatures and clinicopathological features. PLoS ONE. (2008) 3:e3088. 10.1371/journal.pone.0003088 18769486 PMC2518524

[63] Aref D Moffatt CJ Agnihotri S Ramaswamy V Dubuc AM Northcott PA. Canonical TGF-beta pathway activity is a predictor of SHH-driven medulloblastoma survival and delineates putative precursors in cerebellar development. Brain Pathol. (2013) 23:178–91. 10.1111/j.1750-3639.2012.00631.x 22966790 PMC8029114

[64] Nageswara Rao Wallace DJ Billups C Boyett JM Gajjar A Packer JR. Cumulative cisplatin dose is not associated with event-free or overall survival in children with newly diagnosed average-risk medulloblastoma treated with cisplatin based adjuvant chemotherapy: report from the Children's Oncology Group. Pediatr Blood Cancer. (2014) 61:102–6. 10.1002/pbc.24670 23956184 PMC4591537

[65] Zhan M Sun X Liu J Li Y Li Y He X. Usp7 promotes medulloblastoma cell survival and metastasis by activating Shh pathway. Biochem Biophys Res Commun. (2017) 484:429–34. 10.1016/j.bbrc.2017.01.144 28137592

